# Evaluating conversion from mild cognitive impairment to Alzheimer’s disease with structural MRI: a machine learning study

**DOI:** 10.1093/braincomms/fcaf027

**Published:** 2025-01-21

**Authors:** Daniela Vecchio, Federica Piras, Federica Natalizi, Nerisa Banaj, Clelia Pellicano, Fabrizio Piras

**Affiliations:** Neuropsychiatry Laboratory, Department of Clinical Neuroscience and Neurorehabilitation, IRCCS Santa Lucia Foundation, Rome 00179, Italy; Neuropsychiatry Laboratory, Department of Clinical Neuroscience and Neurorehabilitation, IRCCS Santa Lucia Foundation, Rome 00179, Italy; Neuropsychiatry Laboratory, Department of Clinical Neuroscience and Neurorehabilitation, IRCCS Santa Lucia Foundation, Rome 00179, Italy; Department of Psychology, ‘Sapienza’ University of Rome, Rome 00185, Italy; PhD Program in Behavioral Neuroscience, Sapienza University of Rome, Rome 00161, Italy; Neuropsychiatry Laboratory, Department of Clinical Neuroscience and Neurorehabilitation, IRCCS Santa Lucia Foundation, Rome 00179, Italy; Neuropsychiatry Laboratory, Department of Clinical Neuroscience and Neurorehabilitation, IRCCS Santa Lucia Foundation, Rome 00179, Italy; Neuropsychiatry Laboratory, Department of Clinical Neuroscience and Neurorehabilitation, IRCCS Santa Lucia Foundation, Rome 00179, Italy

**Keywords:** Alzheimer prediction, MRI, machine learning

## Abstract

Alzheimer’s disease is a disabling neurodegenerative disorder for which no effective treatment currently exists. To predict the diagnosis of Alzheimer’s disease could be crucial for patients’ outcome, but current Alzheimer’s disease biomarkers are invasive, time consuming or expensive. Thus, developing MRI-based computational methods for Alzheimer’s disease early diagnosis would be essential to narrow down the phenotypic measures predictive of cognitive decline. Amnestic mild cognitive impairment (aMCI) is associated with higher risk for Alzheimer’s disease, and here, we aimed to identify MRI-based quantitative rules to predict aMCI to possible Alzheimer’s disease conversion, applying different machine learning algorithms sequentially. At baseline, T1-weighted brain images were collected for 104 aMCI patients and processed to obtain 146 volumetric measures of cerebral grey matter regions [regions of interest (ROIs)]. One year later, patients were classified as converters (aMCI-c = 32) or non-converters, i.e. clinically and neuropsychologically stable (aMCI-s = 72) based on cognitive performance. Feature selection was performed by random forest (RF), and the identified seven ROIs volumetric data were used to implement support vector machine (SVM) and decision tree (DT) classification algorithms. Both SVM and DT reached an average accuracy of 86% in identifying aMCI-c and aMCI-s. DT found a critical threshold volume of the right entorhinal cortex (EC-r) as the first feature for differentiating aMCI-c/aMCI-s. Almost all aMCI-c had an EC-r volume <1286 mm^3^, while more than half of the aMCI-s patients had a volume above the identified threshold for this structure. Other key regions for the classification between aMCI-c/aMCI-s were the left lateral occipital (LOC-l), the middle temporal gyrus and the temporal pole cortices. Our study reinforces previous evidence suggesting that the morphometry of the EC-r and LOC-l best predicts aMCI to Alzheimer’s disease conversion. Further investigations are needed prior to deeming our findings as a broadly applicable predictive framework. However, here, a first indication was derived for volumetric thresholds that, being easy to obtain, may assist in early identification of Alzheimer’s disease in clinical practice, thus contributing to establishing MRI as a useful non-invasive prognostic instrument for dementia onset.

## Teaser

Alzheimer’s disease lacks effective treatment, and current biomarkers are sub-optimal. Vecchio *et al*. developed MRI-based methods to predict Alzheimer’s disease from amnestic mild cognitive impairment using machine learning. Algorithms achieved 86% accuracy, with a threshold volume >1286 mm³ in the right entorhinal cortex typifying clinical stability.

## Graphical Abstract

**Graphical Abstract:**
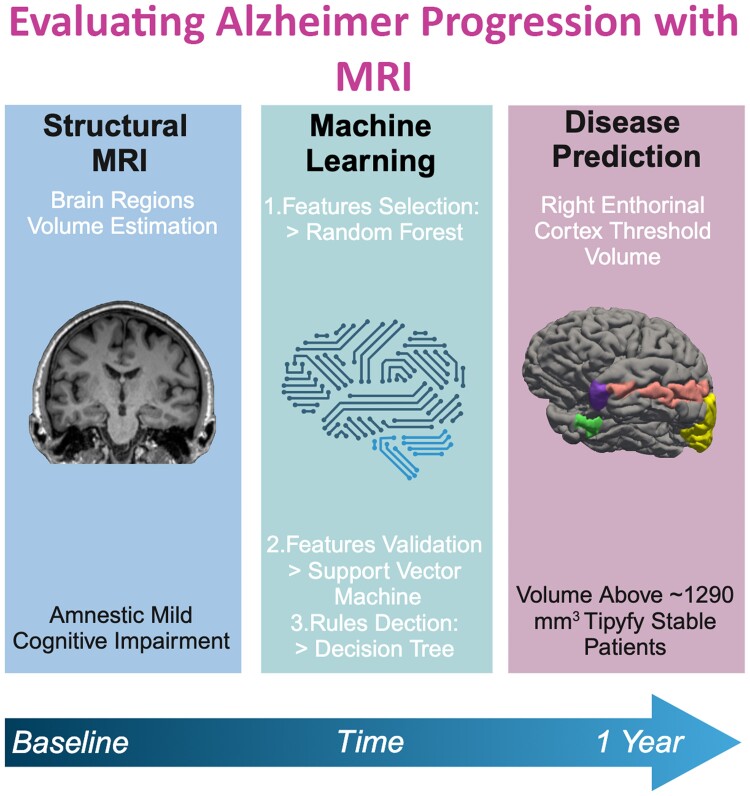

## Introduction

Alzheimer’s disease is a disabling neurodegenerative disease and the most common type of dementia worldwide.^1^ Despite decades of scientific efforts, effective treatments for Alzheimer’s disease are still lacking, and early detection of Alzheimer’s disease is critical for patients’ clinical management and personalized therapy. Indeed, a timely diagnosis and an accurate prognosis of Alzheimer’s disease may enable or encourage more fine-grained lifestyle changes, neurocognitive enrichment and non-pharmacological interventions in pre-clinical stages, to slow down cognitive decline.^2^ Mild cognitive impairment (MCI) is an intermediate stage between normal ageing and Alzheimer’s disease characterized by both an abnormal cognitive decline for the individual’s age and educational level^3^ and relatively spared functions in everyday activities. Detection of Alzheimer’s disease biomarkers is important in MCI and especially in the amnesic sub-type (aMCI), which presents a conversion rate between 8 and 15%^4,5^ per year, and around 50% within 5 years.^6^ However, although promising studies suggest that a reasonably precise prediction of disease occurrence can be achieved by modelling a combination of plasma biomarkers linked to Alzheimer’s disease pathology,^7^ the identification of current Alzheimer’s disease biomarkers in MCI is invasive, time consuming and/or expensive, limiting their application to mass screening and primary care settings.

MRI represents a less-invasive tool, being safe, quick and relatively affordable to perform. Since Alzheimer’s disease brain changes occur even before its clinical manifestation,^8,9^ MRI techniques could be employed in MCI to obtain cerebral morphometric features and to investigate structural biomarkers of disease progression. Specifically, it has been shown that structural brain changes in the temporal cortices,^10^ and especially in the hippocampus,^11^ may act as potential indices of neurodegenerative damage and disease progression in Alzheimer’s disease patients.^12,13^

A substantial body of neuroimaging studies has developed statistical paradigms and machine learning (ML) algorithms to produce more refined diagnostic and prognostic models in the neurology field. ML offers a systematic approach to developing sophisticated, automatic and objective classification frameworks for analysing high-dimensional data, by learning complex patterns of subtle brain changes.^14^ Specifically, the ML paradigm consists in training an algorithm to extract from a given dataset (e.g. brain volumetric measures) common features to classify subjects according to a specified diagnosis. Sequentially, the trained algorithm can be used to make predictions and to categorize different individuals for which the diagnosis is unknown. This research area is currently rapidly advancing, and a recent review showed that ML algorithms can classify MRI images of Alzheimer’s disease, MCI patients and healthy participants [Healthy Controls (HC)] with very high accuracy levels.^15^ Some of these algorithms are also able to distinguish between patients with MCI that will convert (MCI-c) or not (stable MCI, MCI-s) to Alzheimer’s disease, within a defined period of time (*ibidem*). However, these studies are characterized by high methodological heterogeneity such as samples included in the studies, pre-processing techniques, types of neuroimaging data, classification and validation methods. Regarding the samples, most studies use the publicly available data from the Alzheimer’s Disease Neuroimaging Initiative database, which offers the possibility to obtain a large experimental sample size. However, images are characterized by high variability in terms of MRI field strength (i.e. 1.5 and 3 Tesla) and image parameters. Additionally, to be clinically useful and to prevent potential biases by ensuring data diversity to promote generalization, these ML models have to be tested not only in large samples, but also in more variable and diverse groups of clinical populations. Regarding the ML methods applied to classify patients and to detect MCI to possible Alzheimer’s disease progression, the most popular are those based on support vector machine (SVM). This is a supervised algorithm that has demonstrated its utility in neuroimaging-based applications, especially for the classification of future clinical outcomes.^16^ Other commonly used classifiers are random forest (RF) and neural networks, and the latter often relies on convolutional neural networks for images classification tasks.

Key cortical areas identified by such computational models for the discrimination between Alzheimer’s disease patients and MCI or HC are mainly located in the temporal, parietal and frontal lobes. In particular, the hippocampus, amygdala, entorhinal cortex, precuneus, cingulate gyrus, and the rostral and caudal areas of the medial frontal lobe are the most relevant regions for subjects’ classification^17-24^. However, previous studies only reported statistics for model accuracy, sensitivity, specificity or a single scalar value expressing the overall performance of the binary classifier [i.e. the receiver operating characteristic area under curve (ROC-AUC)]. These measures are useful to evaluate the algorithms’ classification performance but are difficult to use for clinical purposes. To the best of our knowledge, previous works have not shown the specific criteria used by the model to classify subjects as aMCI-c or aMCI-s, such as a potential critical threshold value for differentiating these two groups according to atrophy rates.

By sequentially applying different ML algorithms, the present study aims to identify critical values of several cortical and sub-cortical cerebral grey matter volumes that are easy to obtain and to apply in clinical practice, to differentiate aMCI patients who will (aMCI-c) or will not (aMCI-s) convert in possible Alzheimer’s disease within 1 year.

## Materials and methods

### Study sample and clinical assessment

At the Memory Clinic Outpatient Service of IRCCS Santa Lucia Foundation, we initially evaluated 130 aMCI patients (either single or multiple domain) reporting a decrease in their cognitive abilities. In order to be considered as diagnostic evidence,^25^ such changes had to be confirmed by a reliable caregiver, defined as a person contacting the patient weekly, with at least one personal visit. At baseline, all participants were assessed using the Clinical Dementia Rating (CDR)^26^ and Instrumental Activities of Daily Living (IADL)^27^ scales, the Mental Deterioration Battery^28^ (MDB) and other standardized cognitive tests. Specifically, patients were tested for general cognitive functioning [Mini-Mental State Examination^29^ (MMSE)] and different cognitive domains, such as: (i) verbal memory (MDB Rey’s 15-word Immediate Recall and Delayed Recall); (ii) logical reasoning (MDB Raven’s Progressive Matrices 47); (iii) language (MDB Semantic and Phonological Verbal Fluency and MDB Sentence Construction); (iv) simple constructional praxis (MDB Copying Drawings and MDB Copying Drawings with Landmarks); (v) long-term visual memory (Delayed Recall of Rey–Osterrieth picture)^30-32^; (vi) complex constructional praxis (Copy of Rey–Osterrieth picture)^30-32^ and (vii) executive abilities, i.e. attentive shifting and control (Trail Making Test A-B^33^ and Stroop test^34^). Patients’ medical information regarding age of cognitive decline onset, disease duration and treatment intake for ageing-associated diseases like hypertension, hypercholesterolaemia and diabetes was also collected.

Specific inclusion criteria were as follows: (i) age between 55 and 90 years, (ii) vision and hearing sufficient for undergoing the testing procedures, (iii) compliance with assessment procedures and (iv) diagnostic evidence of aMCI (either single or multiple domain) consistent with Petersen’s guidelines^35^ and the following clinical criteria proposed by the MCI Key Symposium 2003^36^: (i) abnormal memory function for age, (ii) preserved autonomy in daily living (≥5), (iii) absence of dementia (MMSE score >20, CDR ≤ 0.5) and (iv) suitability for MRI examination.

Exclusion criteria were as follows: (i) major medical illnesses, (ii) primary psychiatric or neurological disorders comorbidity, (iii) history of alcohol or drug abuse and (iv) MRI evidence of focal parenchymal abnormalities or neoplasm.

After 1 year, each patient was assessed in a follow-up visit, and the same diagnostic procedures were administered to confirm the baseline aMCI diagnosis or to attest the aMCI to Alzheimer’s disease conversion status, based on clinical criteria. According to the National Institute of Neurological and Communicative Diseases and Stroke/Alzheimer's Disease and Related Disorders Association (NINCDS-ADRDA) criteria,^37^ the diagnosis of possible Alzheimer’s disease was made for patients with a CDR score >0.5 combined either with a MMSE score ≤20 or with a neuropsychological performance below the outer tolerance limit for the reference population in two or more domains, including memory.^38^

Patients were labelled as aMCI-c or aMCI-s respectively whether they meet or not criteria for possible Alzheimer’s disease at the 1-year follow-up clinical evaluation. The 1-year interval was chosen because it is long enough to capture structural changes that develop slowly and gradually over time, being at the same time appropriate for prognostic purposes.

From the initial sample, 26 patients were excluded from the study due to drop-out before the 1-year follow-up (*n* = 18), severe MRI motion artefact (*n* = 5) or evidence of severe white matter lesions (*n* = 3). The final sample included 104 aMCI patients, resulting in 32 aMCI-c and 72 aMCI-s patients.

The study was approved by the local ethics committee. Written informed consent was obtained after presentation of the nature and purposes of the study, from both patients and their informants.

### MRI acquisition and processing

All participants underwent the same imaging protocol at baseline, acquired using a 3T Allegra MR imager (Siemens, Erlangen, Germany) and a standard quadrature head coil. The protocol included 3D whole-brain high-resolution T1-weighted (T1-w), T2-weighted (T2-w) and fluid-attenuated inversion recovery (FLAIR) images. All planar sequences were obtained in the plane of the anterior commissure–posterior commissure (AC–PC) line. Care was taken to centre the subjects in the head coil and to restrain their movements with cushions and adhesive medical tape. Whole-brain T1-w images were obtained in the sagittal plane using a modified driven equilibrium Fourier transform (Time to Echo = 2.4 ms, Repetition Time = 7.92 ms, flip angle = 15° and voxel-size = 1 × 1 × 1 mm). T2-w and FLAIR sequences were acquired to screen for brain pathology.

T1-w images were used to obtain volumes of cortical and sub-cortical regions of interest (ROIs). To optimize signal-to-noise ratio, images were initially corrected for spatially varying noise levels and intensity inhomogeneity, using a validated adaptive non-local means denoising software.^39^ Denoised images were then processed with the recon-all standard pipeline implemented in FreeSurfer version v.7.1.1 (available online), resulting in 68 bilateral cortical regions and 14 bilateral deep grey matter structures (i.e. hippocampi, thalami, amygdala, putamen, caudate, accumbens and pallidum), according to the Desikan–Killiany parcellation atlas.^40^ In order to avoid excluding region not properly segmented (i.e. avoid missing data), the cortical/sub-cortical ROIs output from the recon-all step were one-by-one visually inspected and manually corrected in terms of regions filling/emptying, in case they were under/overestimated by the software. Manual corrections were performed for almost all participants by a PhD student trained in neuroimaging and blinded to the study aim. Cortical/sub-cortical volumes were then extracted from the corrected images, using the FreeSurfer mri_segstats tool.

Dedicated FreeSurfer segmentation modules were additionally applied to the corrected T1-w images to obtain volumes of the hippocampal^41^ sub-fields and amygdala^42^ and thalamic^43^ sub-nuclei.

A total of 146 brain volumes (features) including 68 cortical, 8 sub-cortical, 38 hippocampal, 18 amygdala and 14 thalamic measures were thus extracted from the MRI processing (Supplementary Table 1).

**Figure 1:**
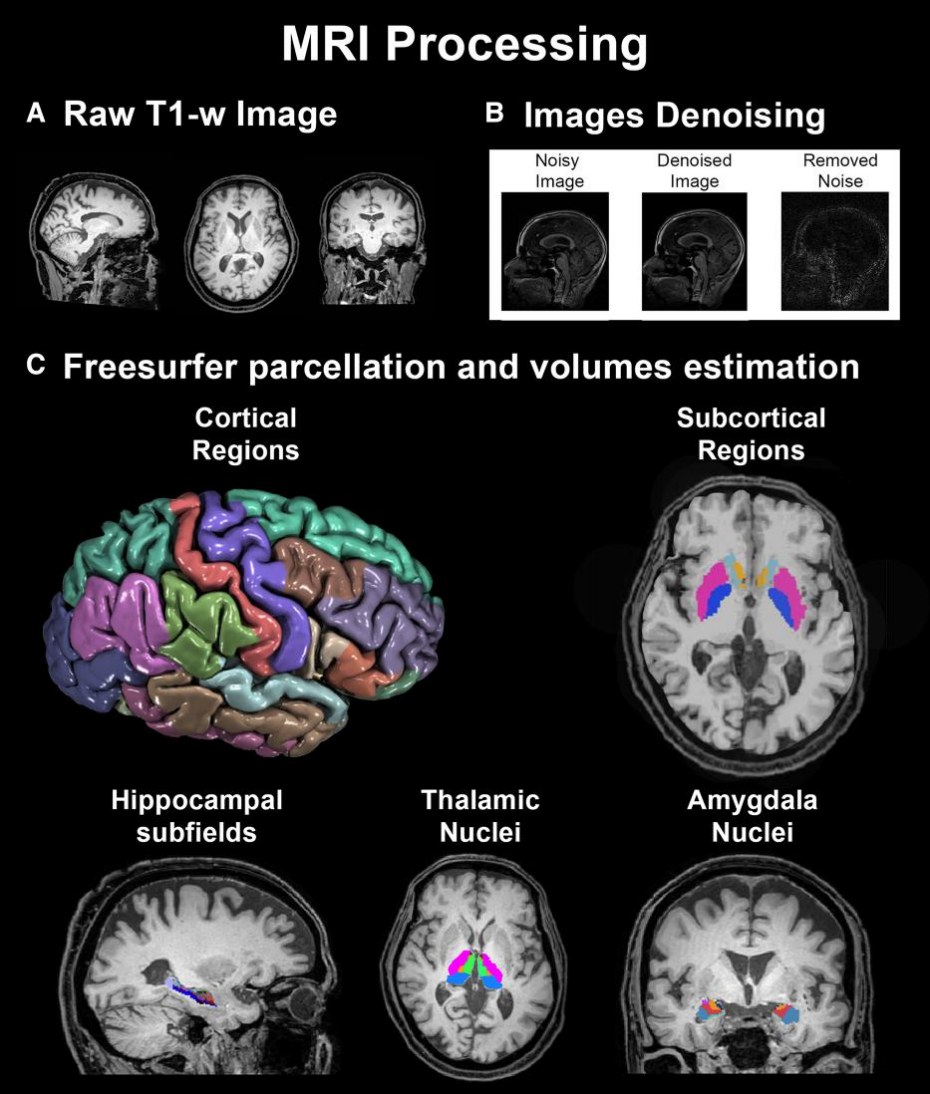
**T1-w images processing.** Workflow of MRI processing: (**A**) 3D T1-w images were collected in 104 aMCI patients; (**B**) each image was denoised to increase the signal-to-noise ratio; (**C**) cortical and sub-cortical brain regions and hippocampal sub-fields, thalamic and amygdala nuclei were segmented using FreeSurfer software, to obtain volumetric measures of 146 brain regions of interest. Colour legend: Sub-cortical regions: ▪ medium violet red: putamen nucleus; ▪ fersian blue: pallidum nucleus; ▪ yacht blue: caudate nucleus; ▪ golden rod: accumbens nucleus. Cortical regions: ▪ deep indigo: lateral occipital; ▪ green sage: fusiform; ▪ medium orchid: inferior parietal; ▪ corrosion green: superior parietal; ▪ flat green: supramarginal; ▪ seawater: superior temporal; ▪ fudge: middle temporal; ▪ purple zergling: inferior temporal; ▪ ant red: post-central; ▪ waiporoporo purple: pre-central; ▪ spearmint: superior frontal; ▪ carob brown: caudal middle frontal; ▪ arabian silk: rostral middle frontal; ▪ weathered Wood: pars triangularis; ▪ terracotta: pars opercularis; ▪ dark sorrel: pars orbitalis. Hippocampal sub-fields: ▪ cobalite: hippocampal tail; ▪ blue: subiculum; ▪ cavalry brown: molecular layer body; ▪ arterial blood red: molecular layer head; ▪ whiplash: CA1 head; ▪ green: CA3 body; ▪ kabocha green: CA3 head; ▪ dune drift: CA4 body; ▪ arable brown: CA4 head; ▪ bright indigo: hippocampal fissure; ▪ turquoise: GC-ML-DG body; ▪ sugar paper: GC-ML-DG head. Thalmic nuclei: ▪ neon pink: ventral nucleus; ▪ malachite green: anterior nucleus (anteroventral); ▪ bright light green: medial nucleus; ▪ clementine: intra-laminar nucleus; ▪ blue nebula: posterior nucleus. Amygdala nuclei: ▪ athens: lateral nucleus; ▪ infrared flush: basal nucleus; ▪ orange brown: accessory basal nucleus; ▪ navy blue: cortical amygdaloid transition; ▪ violet: central nucleus; ▪ sacro bosco: medial nucleus; ▪ creamy avocado: cortical nucleus; ▪ carribean green: paralaminar nucleus.

Finally, total intra-cranial volume was extracted from the FreeSurfer segmentation procedure and used to correct regional volumes for variations in global brain size. The normalized regional volumes were then multiplied by 10^7^, to restore their natural scale of values. See Fig. 1 for the whole MRI processing representation.

#### Statistical analyses

Differences between aMCI-c and aMCI-s in baseline demographic (age, gender and educational level) and clinical (age of cognitive decline onset, illness duration and pharmacotherapy) characteristics were evaluated using Student’s *t* or χ^2^ tests. Statistics were performed using SPSS Statistics version 25.0 (IBM, Armonk, NY, USA), considering *P* < 0.05 as the statistical threshold for significance.

#### Machine learning procedure

To pursue the study aim, three different scikit-learn ML algorithms as implemented in Python 3.8 were sequentially used, using 80% of the dataset for training the algorithms and the remaining for testing. Specifically, RF, SVM and decision tree (DT) models were used to perform, respectively, features selection, features validation and classification rules detection. RF fits several trees classifiers on various sub-samples of the dataset and uses averaging to improve the predictive accuracy and control over-fitting. SVM is a powerful supervised ML model that uses classification algorithms for two-group classification problems on small but complex datasets. DT is a supervised learning algorithm that uses a hierarchical tree structure for classification, providing easy-to-understand models. Its structure consists of a root node, branches, internal nodes and leaf nodes. Each node in the tree tests a condition on an attribute, and each branch descending from that node corresponds to one of the possible values for that attribute condition (i.e. true/false).

**Figure 2:**
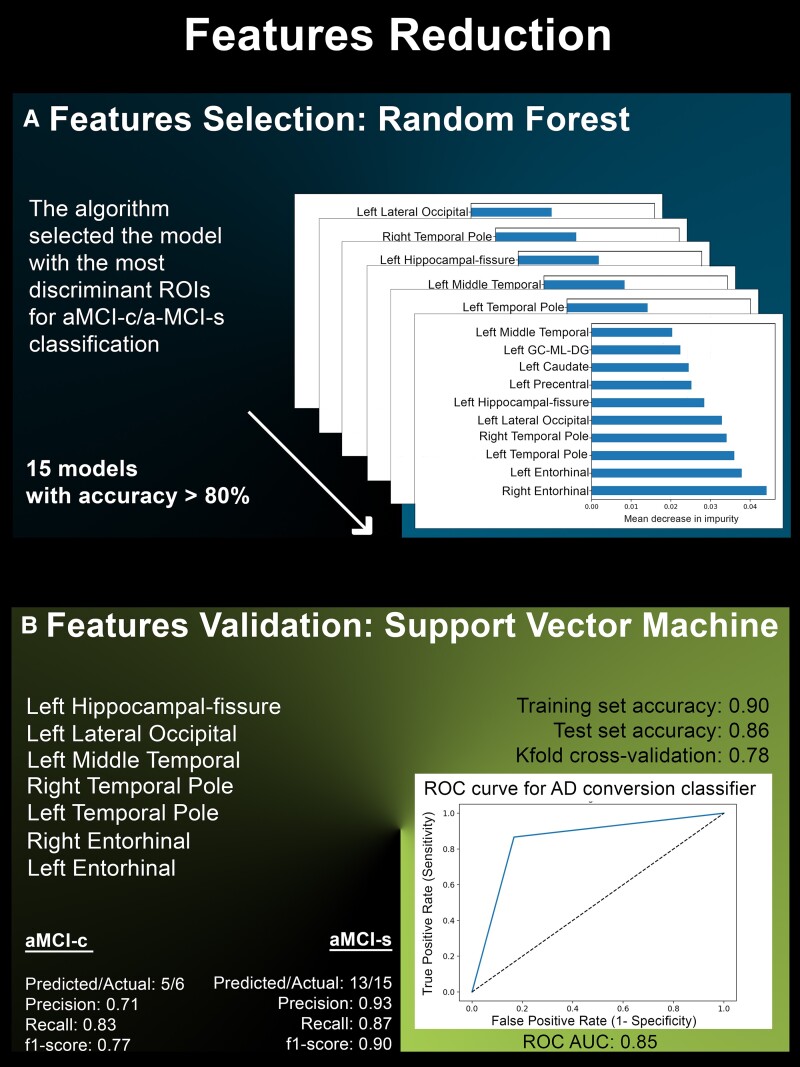
**Features reduction.** (**A**) Random forest models, applied for features selection to reach the amount of 15 models with accuracy >80%. (**B**) Support vector machine model, applied to validate features selected by random forest models. GC-ML-DG, granule cell/molecular layers of the dentate gyrus.

**Figure 3:**
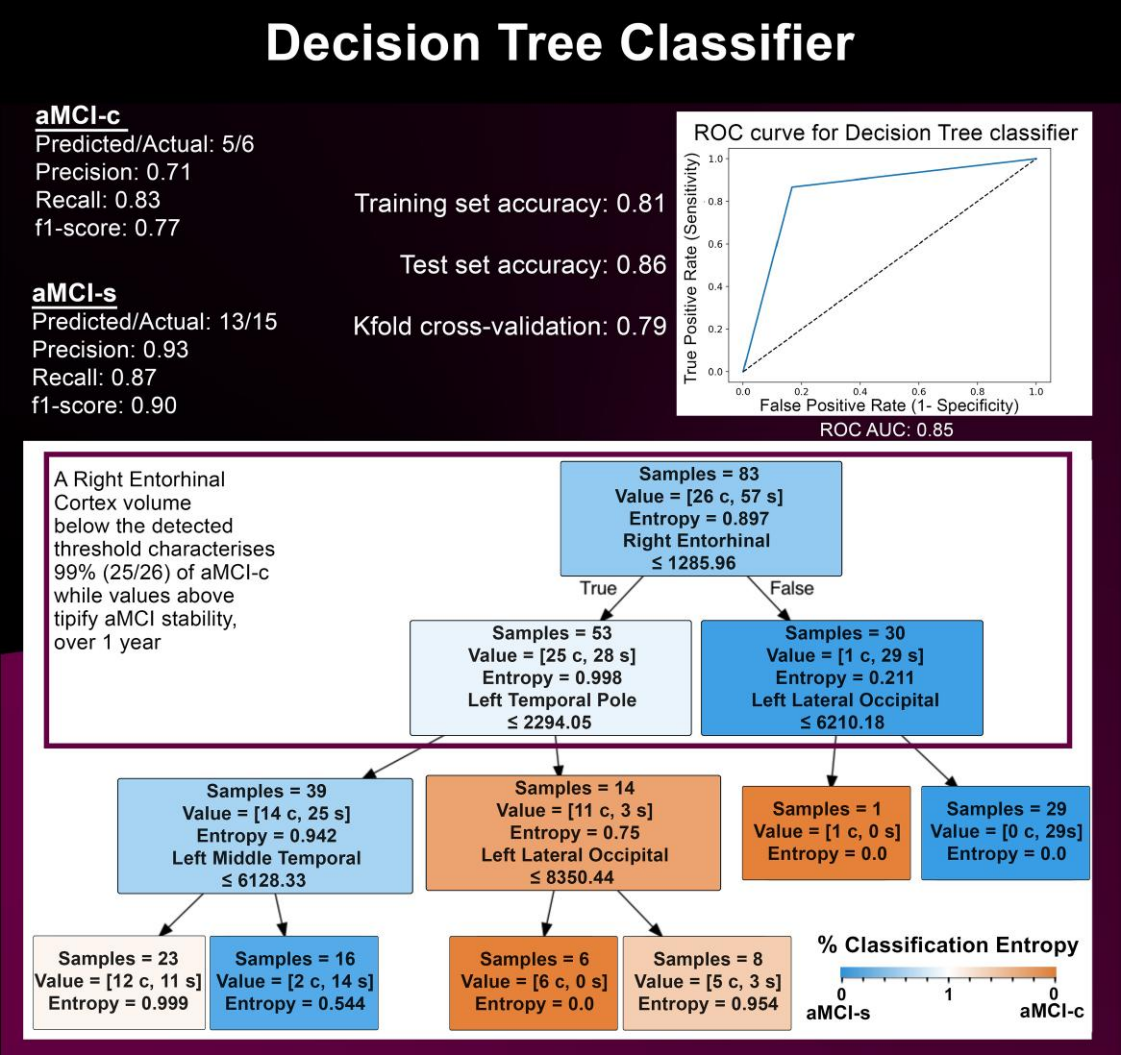
**Decision tree classifier.** DT model, applied for rules detection and to identify thresholds volumes used to classify amci-c and amci-s patients.

For each described ML algorithm, the confusion matrix, models precision (precision = true positive/predicted positive), recall ability (recall = true positive/actual positive) and the f1-score [f1 = 2 × (precision × recall/precision + recall)] were computed to evaluate models’ accuracy. As for SVM and DT, the models sensitivity and specificity were further assessed by computing the ROC-AUC and the cross-validated ROC-AUC. Finally, the *k*-fold method (dividing the dataset into *k* subsets and training/testing the model *k* times) was used as a model cross-validation procedure, using *k* = 100 (Figs. 2 and 3).

### Features selection

To prevent over-fitting, reduce model complexity and avoid multi-collinearity or noising information among features, a features selection procedure was performed using RF algorithms. Specifically, the RF algorithms were implemented to select the 10 most discriminant features for the classification purpose. The threshold of 10 features was chosen according to the rule of *n*_features_ = √*n*_cases_ for correlated measures.^44^ The RF classifier was set with 100 as the number of estimators, ‘entropy’ as the classification criteria and 3 as the maximum depth of the trees. The procedure was recursively repeated until a number of 15 models with a total accuracy >80% were reached, and features were selected only if involved in at least 50% of the 15 models.

To investigate the involvement of the hippocampal sub-fields, thalamic and amygdala sub-nuclei or their whole structure into the diagnostic classification, two different datasets were sequentially used for the features selection process. Specifically, the features selection procedure was initially performed using the most detailed and parcelled dataset, which included each volume for the individual sub-fields/sub-nuclei of the bilateral hippocampi, amygdala and thalami. Total volumes of the bilateral thalami, amygdala and hippocampi were thus excluded from this first dataset, to avoid the presence of redundant features. In case none of the amygdala and thalamic sub-nuclei resulted among the 10 most discriminating features identified by the RF classification procedure, they were sequentially merged as global structures measures. Hippocampal sub-fields were merged into head, body, tail and fissure sub-fields before being considered as whole structure volume. The final dataset comprised a total of 104 brain features as result from the sub-fields/sub-nuclei merging procedure (see Supplementary Table 1 for a detailed ROIs list), and it was used to re-run the selection procedure as previously described.

### SVM model

An SVM model was used to further assess the suitability and accuracy of the selected brain features for the diagnostic classification task. To improve the SVM performance, multiple hyper-parameters tuning were evaluated using the Grid Search Python tool. Specifically, the latter was implemented to find the best combination of regularization parameter (C), kernel type (kernel), degree of the kernel function (degree) and kernel coefficient (gamma) to be used in the SVM algorithm, using 10-fold cross-validation dataset splits.

### Decision tree model

To move beyond statistics regarding ML models classification accuracy and to find brain critical threshold volumes as classification rules to predict the aMCI-c or aMCI-s patients’ status at follow-up, a DT model was implemented using the selected features. The DT classifier was run with the ‘entropy’ criteria and 3 as the maximum depth of the tree.

## Results

### Sample characteristics

No group differences were found between aMCI-c and aMCI-s in baseline demographic (age, gender and educational level) and clinical (age of cognitive decline onset, illness duration, pharmacotherapy and IADL scores) characteristics, with the exception of the CDR score (Table 1). Clearly, at the follow-up assessment, the two groups were significantly different in terms of CDR and IADL scores, as well as in their cognitive profile (MMSE score and the number of impaired domains).

**Table 1:** Demographic and clinical characteristics of aMCI-c and aMCI-s groups

	aMCI-c (*n* = 32)	aMCI-s (*n* = 72)	*t*, χ^2^	df	*P*
Age, mean (SD)	75.5 (7.5)	73.93 (6.19)	−1.12	102	0.27
Gender, male (%)	13 (41%)	39 (54%)	1.625	1	0.2
Years of education, mean (SD)	11.28 (5.35)	9.93 (4.79)	−1.28	102	0.2
Age of onset, mean (SD)	72.73 (7.95)	70.86 (5.91)	−1.28	92	0.2
Months of illness, mean (SD)	30.9 (30.8)	30.8 (28.1)	0.02	92	0.98
IADL, mean (SD)	5.84 (1.6)	5.81 (1.4)	0.121	102	0.9
IADL f.u., mean (SD)	4.53 (1.9)	5.5 (1.4)	−2.78	102	0.007
CDR, mean (SD)	0.48 (0.09)	0.38 (0.21)	2.61	102	0.01
CDR f.u., mean (SD)	1.1 (0.25)	0.36 (0.22)	14.23	102	<0.0001
Number of impaired domains, mean (SD)	3.5 (1.6)	2.5 (1.4)	3.09	102	0.003
Number of impaired domains f.u., mean (SD)	4.9 (1.4)	2.3 (1.4)	7.11	102	<0.0001
MMSE, mean (SD)	25.69 (1.89)	26.49 (2.1)	1.85	102	0.07
MMSE f.u., mean (SD)	23.38 (3.8)	26.68 (2.33)	5.4	101	<0.0001
Treated hypertension, yes (%)	11 (48%)	33 (63%)	1.608	1	0.2
Treated diabetes, yes (%)	2 (9%)	8 (15%)	0.617	1	0.43
Treated hypercholesterolaemia, yes (%)	12 (52%)	24 (46%)	0.232	1	0.63

IADL, Instrumental Activity of Daily Life Scale; f.u., follow-up visit; df, degrees of freedom; *n*, number of subjects; SD, standard deviation; t, Student’s *t*-test.

### Features selection

Among the 104 ROIs of the final database, 7 were selected by the features selection procedure, namely the left hippocampal fissure (which was discriminant in 8 out of 15 models—8/15), the left lateral occipital (9/15), the left middle temporal (9/15) cortices, the right and left temporal pole (9/15 and 12/15, respectively) and the right and left entorhinal cortices (14/15 and 15/15, respectively; Fig. 2).

### Support vector machine model

The hyper-parameter tuning performed by Grid Search showed that the SVM achieved the best results in 10-fold cross-validated dataset splits by combining C = 1000, degree = 3, gamma = 0.03 and kernel = polynomial parameters. Using these parameters and the seven selected ROIs, the SVM model classified aMCI-c and aMCI-s with a total accuracy of 90% for the training dataset and 86% for the test dataset, indicating a low risk of over-fitting. The model precision was 93% for aMCI-s and 71% for aMCI-c; the recall was 87% for aMCI-s and 83% for aMCI-c. Accordingly, the f1-score (namely, the harmonic mean between the ratio of true positives to total predicted positive and the fraction of correctly predicted positive to all actual positives, which balances precision and recall values) was 90% for aMCI-s and 77% for aMCI-c. The computed ROC-AUC was 85%, while the cross-validated ROC-AUC was 80%. Finally, the *k*-fold cross-validation procedure showed an average stratified cross-validation accuracy score of 78% (Fig. 2).

### Decision tree model

The DT model reached almost the same accuracy, sensitivity and specificity of the SVM one. Specifically, the model classified aMCI-c and aMCI-s with a total accuracy of 81% for the training dataset and 86% for the test dataset, indicating a low over-fitting risk. The model’s precision was 93% for aMCI-s and 71% for aMCI-c classification; the recall was 87% for aMCI-s and 83% for aMCI-c. Accordingly, the f1-score was 90% for aMCI-s and 77% for aMCI-c. The computed ROC-AUC was 85%, while the cross-validated ROC-AUC was 69%. Finally, the *k*-fold cross-validation procedure showed an average stratified cross-validation accuracy score of 79%.

The volume of the right entorhinal cortex (EC-r) was the first feature allowing the binary classification of aMCI-c or aMCI-s patients, since almost all aMCI-c (25 out of 26 patients) had an EC-r volume below the threshold of 1286 mm^3^ (Fig. 3). Considering the total of 83 patients included in the training dataset, the model was not able to identify specific rules to perfectly classify the two groups in order to obtain a class consisting only of the 26 aMCI-c patients and a class consisting only of the 57 aMCI-s patients (i.e. the two complete groups with class entropy = 0, i.e. perfect homogeneity of the elements within the class). Indeed, the most homogeneous classification for aMCI-c included six patients showing an EC-r volume below threshold and a left lateral occipital cortex (LOC-l) volume smaller than a threshold of 8350 mm^3^. The remaining aMCI-c patients, who were not categorized according to the EC-r and LOC-l, were classified considering threshold values for the left temporal pole and left middle temporal gyrus atrophy, but with a higher level of class entropy.

About half of the aMCI-s patients showed an EC-r volume above 1286 mm^3^ and a LOC-l >6210 mm^3^, representing the most homogeneous aMCI-s group (class entropy = 0) and including 29 out of the total 57 patients. Most aMCI-s patients who did not meet the criteria of an EC-r larger than 1286 mm^3^ also showed a small left temporal pole volume, possibly indicating that the EC-r atrophy is more widespread in this aMCI-s sub-group, thus encompassing additional cortical areas.

## Discussion

This work was carried out to identify critical volumes of different cortical and sub-cortical cerebral grey matter regions differentiating stable aMCI patients from aMCI patients who convert to possible Alzheimer’s disease, within a 1-year timeframe. The principal aim was to find out brain measures easy to obtain and to be possibly used in clinical practice. We found that 7 of the total 146 investigated brain regions (specifically, the bilateral entorhinal and temporal pole cortices, the left middle temporal gyrus, the LOC-l and the left hippocampal fissure) are crucial for the aMCI-c/aMCI-s binary classification process. Moreover, the main result of this work was that a volume of the EC-r below the threshold value of 1286 mm^3^ characterizes 99% of the aMCI-c, while values above typify the longitudinal stability of the clinical status.

All the cortical regions that resulted as discriminant for aMCI-c/aMCI-s classification in our study were already well known to be involved in Alzheimer’s disease–related neurodegeneration.^11,45-47^ However, to the best of our knowledge, our ML method is the first to suggest precise threshold volumes of specific brain regions for the biomarker-based prognosis of aMCI conversion (or not) in possible Alzheimer’s disease, within 1 year.

Previous evidence suggests that early pathological changes of Alzheimer’s disease occur in the EC,^11,45-47^ which has been highlighted as a valuable biomarker in MCI when predicting future conversion to Alzheimer’s disease, especially for the right hemisphere.^45^ The EC is a nodal point in the cortico-hippocampal network, and evidence demonstrated its critical role in navigation, a cognitive ability underpinned by the firing of spatially modulated neurons within the EC (i.e. grid cells) and specifically impaired in MCI.^48^ The entorhinal–hippocampal circuit is crucially involved in memory formation and retrieval, and damage to this circuit results in memory impairments^49^ typically observed in MCI.^50^ Recent histological and neuroimaging findings suggest that neurons in the EC Layer II are specifically vulnerable to degeneration in Alzheimer’s disease, showing early functional vulnerability that precedes neurodegeneration.^51^ Moreover, post-mortem studies have revealed that neurofibrillary tangles originates in the EC Layer II,^11,46,47^ causing the neuronal loss observed in individuals with MCI even before the onset of Alzheimer’s disease.^52^ Although the underlying mechanisms are still under intense investigation, a recent review^53^ proposed a detailed model to explain the molecular mechanisms of the earliest pathological events in EC Layer II. The model proposes that age-related disruptions in mitochondrial homeostasis cause the build-up of intra-cellular Aβ oligomers in certain neurons. These neurons have high metabolic demands due to their intensive integration efforts, leading to reduced tolerance for partial impairments. The Aβ intra-cellular accumulation hinders efficient signalling, starting the cascade leading to protein Tau misfolding and the accumulation of neurofibrillary tangles. This process may enhance the deposition of pathological proteins in synaptically linked neurons in the pre-clinical stage of Alzheimer’s disease, ultimately playing a role in the progression of the disease (*ibidem)*.

Several longitudinal studies^54-56^ showed that patients with cognitive complaints converting to Alzheimer’s disease exhibited a significant volume loss in the EC. This prognostic value of the EC atrophy was also validated by the ERICA tool,^57^ an MRI-based visual rating scale that uses scores ranging from 0 (no atrophy) to 3 (severe atrophy) to rate EC morphometry. Compared with the ERICA scale, our method overcomes issues implicit in qualitative scales such as inter- and intra-rater variability,^58^ hampering consistency in longitudinal studies.^59^ Moreover, even though our method needs a slighter effortful processing than ERICA, it is rewarded by the better accuracy reached in predicting the clinical outcome of aMCI (sensitivity, specificity and ROC-AUC of ERICA scale were respectively 53, 86 and 70%^57^).

Taken together, our results enhance this evidence of the EC-r volume as a significant predictor of the likelihood of Alzheimer’s disease conversion, also identifying a critical threshold for the EC-r volume to characterize aMCI stability. Indeed, patients showing an EC-r volume greater than the detected critical value did not convert to Alzheimer’s disease within 1 year.

It should be noted that half of the aMCI-s patients also showed an EC-r below the threshold volume, possibly suggesting that these individuals may convert over a longer period. This hypothesis should be explored in additional studies, considering a wider time window to verify whether patients with an EC-r volume below the critical threshold may convert in Alzheimer’s disease before those with an EC-r volume above the critical threshold. In any case, the critical volumetric threshold here identified might be useful to monitor aMCI’s annual stability, offering the possibility of early personalized interventions.

Moving forward, we found that 23% of aMCI-c patients are characterized by both a volume below the EC-r critical threshold and a volume lower than 8350 mm^3^ in the LOC-l, with a perfect homogeneity within the sample. Moreover, the only aMCI-c patient with an EC-r volume above the critical threshold was successfully classified according to the LOC-l volume, suggesting the relevance of this cortical structure in predicting Alzheimer’s disease conversion. The lateral occipital (LOC) morphology was already found as implicated in the neurodegenerative process involved in Alzheimer’s disease, and cortical features of aMCI patients in this region resulted in values halfway between those of healthy and Alzheimer’s disease subjects, being significantly different from both.^60^ The LOC is involved in both the ventral and dorsal visual pathways,^61^ and deficits in vision are common in Alzheimer’s disease, either at the ‘lower’ (i.e. contrast sensitivity, visual acuity, colour and motion perception) or ‘higher’ levels of visual processing (i.e. reading, object recognition and spatial localization) (see^62^ for a review). Moreover, the LOC resulted as engaged in either the default mode or the frontoparietal networks^63^ (both functionally impaired in AD^64^), and evidence suggests that amyloid-beta accumulations in occipital lobes can predict an earlier Alzheimer’s disease conversion.^65^ Our results are in line with this evidence, suggesting a key role of the LOC-l volume in predicting Alzheimer’s disease progression.

Before concluding, some limitations need to be considered. First, the aMCI-c group is smaller than the aMCI-s one, possibly leading to lower accuracy of the ML algorithm in classifying the former. However, the conversion rate here reported perfectly aligns with the probability for aMCI to be diagnosed with AD after 12 months, providing our work with high ecological validity. Moreover, ML performances are anyway statistically accurate and reliable in the aMCI-c sample, also revealing cortical regions already known as implicated in Alzheimer’s disease conversion. The second limitation to be discussed deals with the number of longitudinal follow-ups here considered. As mentioned before, additional follow-ups are needed to verify the hypothesis of an early conversion for the aMCI-s group showing an EC-r volume below the threshold. Moreover, our method can be used to stage the prodromal manifestations of Alzheimer’s disease more comprehensively, i.e. including groups of patients with subjective memory complaints^66^ before they progress or not to MCI. However, such experimental design would require the shifting to a cross-sectional study, since monitoring a consistent number of patients for such a long-lasting period may prove to be difficult to achieve in real clinical contexts. Last, the *k*-fold cross-validation methodologies employed in our study should be reinforced through the application of our model on a novel and distinct dataset, in order to conclusively ascertain the generalizability of our results. This represents the objective of our current research’s aim, wherein we are concurrently assembling a new longitudinal dataset utilizing a different MR scanner and involving alternative aMCI patients. Furthermore, we are implementing the identified classification rules within our Memory Clinic Outpatient Service, to substantiate the clinical significance of the determined EC-r threshold. This comprehensive approach not only aims to enhance the robustness of our findings but also seeks to establish a more reliable framework for early diagnosis and intervention in patients with aMCI.

Concluding, even though further investigations are needed to generalize our model, the employment of different and subsequent ML algorithms allows us to identify critical threshold values for EC-r and LOC-l volumes, to aid the prediction of aMCI to possible Alzheimer’s disease conversion. Our study reinforces previous evidence suggesting that the morphometry of such cortical regions best predicts MCI to Alzheimer’s disease conversion and demonstrates that MRI could be a useful non-invasive sensitive tool to predict Alzheimer’s disease progression. Indeed, the measures employed in this work are quite easy to compute and to translate in clinical practice, helping the prognosis of aMCI to possible Alzheimer’s disease conversion in a convenient way.

## Supplementary Material

fcaf027_Supplementary_Data

## Data Availability

Anonymized data not published within this article will be made available by request from any qualified investigator. ML codes are included in the Supplementary Material (S2).
