# Supplementary material for: Evaluating conversion from mild cognitive impairment to Alzheimer’s disease with structural MRI: a machine learning study
Source: Brain Commun. 2025 Jan 21;7(1):fcaf027. doi: 10.1093/braincomms/fcaf027 (PMC11780885; doi:10.1093/braincomms/fcaf027)
Supplement: fcaf027_Supplementary_Data [file fcaf027_supplementary_data.docx]

Supplementary Material

*Supplementary Table 1. List of the extracted brain volumes*

| **Total Extracted Brain Volumes** | | |
| --- | --- | --- |
| **Brain Districts** | **All ROIs *(first database)*** | **Subnuclei/Subfields Merge *(final database)*** |
| **Thalamus Nuclei (N=14)** | L/R Anterior Nucleus | L/R Thalamus |
|  | L/R Intralaminar Nucleus |  |
|  | L/R Lateral Nucleus |  |
|  | L/R Medial Nucleus |  |
|  | L/R Geniculate Nucleus |  |
|  | L/R Pulvinar Nucleus |  |
|  | L/R Ventral Nucleus |  |
| **Amygdala Nuclei (N=18)** | L/R Accessory-Basal Nucleus | L/R Amygdala |
|  | L/R Anterior Amygdaloid-Area |  |
|  | L/R Basal Nucleus |  |
|  | L/R Central Nucleus |  |
|  | L/R Cortical Nucleus |  |
|  | L/R Corticoamygdaloid Transitio |  |
|  | L/R Lateral Nucleus |  |
|  | L/R Medial Nucleus |  |
|  | L/R Paralaminar Nucleus |  |
| **Hippocampal Subfields (N=38)** | L/R CA1-Body | L/R CA1 |
|  | L/R CA1-Head |  |
|  | L/R CA3-Body | L/R CA3 |
|  | L/R CA3-Head |  |
|  | L/R CA4-Body | L/R CA4 |
|  | L/R CA4-Head |  |
|  | L/R Fimbria | L/R fimbria |
|  | L/R GC-ML-DG-Body | L/R GC-ML-DG |
|  | L/R GC-ML-DG-Head |  |
|  | L/R HATA | HATA |
|  | L/R Molecular Layer-Body | L/R Molecular Layer |
|  | L/R Molecular Layer-Head |  |
|  | L/R Parasubiculum | L/R parasubiculum |
|  | L/R Presubiculum-Body | L/R Presubiculum |
|  | L/R Presubiculum-Head |  |
|  | L/R Subiculum-Body | L/R Subiculum |
|  | L/R Subiculum-Head |  |
|  | L/R Hippocampal Tail | L/R Hippocampal Tail |
|  | L/R Hippocampal-Fissure | L/R Hippocampal-Fissure |
| **Subcortical Regions (N=8)** | L/R Accumbens Nucleus | L/R Accumbens Nucleus |
|  | L/R Caudate Nucleus | L/R Caudate Nucleus |
|  | L/R Pallidum Nucleus | L/R Pallidum Nucleus |
|  | L/R Putamen Nucleus | L/R Putamen Nucleus |
| **Cortical  Regions (N=68)** | L/R Banks STS cortex | L/R Banks STS cortex |
|  | L/R Caudal Anterior Cingulate cortex | L/R Caudal Anterior Cingulate cortex |
|  | L/R Caudal Middle Frontal cortex | L/R Caudal Middle Frontal cortex |
|  | L/R Cuneus cortex | L/R Cuneus cortex |
|  | L/R Entorhinal cortex | L/R Entorhinal cortex |
|  | L/R Frontal Pole cortex | L/R Frontal Pole cortex |
|  | L/R Fusiform cortex | L/R Fusiform cortex |
|  | L/R Inferior Parietal cortex | L/R Inferior Parietal cortex |
|  | L/R Inferior Temporal cortex | L/R Inferior Temporal cortex |
|  | L/R Insula cortex | L/R Insula cortex |
|  | L/R Isthmus Cingulate cortex | L/R Isthmus Cingulate cortex |
|  | L/R Lateral Occipital cortex | L/R Lateral Occipital cortex |
|  | L/R Lateral Orbitofrontal cortex | L/R Lateral Orbitofrontal cortex |
|  | L/R Lingual cortex | L/R Lingual cortex |
|  | L/R Medial Orbitofrontal cortex | L/R Medial Orbitofrontal cortex |
|  | L/R Middle Temporal cortex | L/R Middle Temporal cortex |
|  | L/R Paracentral cortex | L/R Paracentral cortex |
|  | L/R Parahippocampal cortex | L/R Parahippocampal cortex |
|  | L/R Pars Opercularis cortex | L/R Pars Opercularis cortex |
|  | L/R Pars Orbitalis cortex | L/R Pars Orbitalis cortex |
|  | L/R Pars Triangularis cortex | L/R Pars Triangularis cortex |
|  | L/R Pericalcarine cortex | L/R Pericalcarine cortex |
|  | L/R Post Central cortex | L/R Post Central cortex |
|  | L/R Posterior Cingulate cortex | L/R Posterior Cingulate cortex |
|  | L/R Precentral cortex | L/R Precentral cortex |
|  | L/R Precuneus cortex | L/R Precuneus cortex |
|  | L/R Rostral Anterior Cingulate cortex | L/R Rostral Anterior Cingulate cortex |
|  | L/R Rostral Middle Frontal cortex | L/R Rostral Middle Frontal cortex |
|  | L/R Superior Frontal cortex | L/R Superior Frontal cortex |
|  | L/R Superior Parietal cortex | L/R Superior Parietal cortex |
|  | L/R Superior Temporal cortex | L/R Superior Temporal cortex |
|  | L/R Supramarginal cortex | L/R Supramarginal cortex |
|  | L/R Temporal Pole cortex | L/R Temporal Pole cortex |
|  | L/R Transverse Temporal cortex | L/R Transverse Temporal cortex |

*L/R: Left/Right; GC-ML-DG: granule cell/molecular layers of the dentate gyrus; HATA: hippocampus-amygdala-transition-area; STS: superior temporal sulcus.*

*S2. Python codes for ML analyses*

Random Forest

import numpy as np

import pandas as pd

import matplotlib.pyplot as plt

from matplotlib.pyplot import figure

import seaborn as sns

from sklearn import preprocessing

from sklearn.preprocessing import LabelEncoder

from sklearn.preprocessing import StandardScaler

from sklearn.model_selection import train_test_split

from sklearn.metrics import classification_report,confusion_matrix

from sklearn.ensemble import RandomForestClassifier

df = pd.read_csv("Data.csv")

df

df.columns

df['DX'].value_counts()

X_Train, X_Test, Y_Train, Y_Test = train_test_split(X, Y, stratify=Y, test_size = 0.20)

trainedforest=RandomForestClassifier(n_estimators=100,criterion='entropy',max_depth=3).fit(X_Train,Y_Train)

predictionforest = trainedforest.predict(X_Test)

print(confusion_matrix(Y_Test,predictionforest))

print(classification_report(Y_Test,predictionforest))

fig, ax = plt.subplots()

ax.set_title("Feature importances using MDI")

ax.set_xlabel("Mean decrease in impurity")

feat_importances = pd.Series(trainedforest.feature_importances_, index= X.columns)

feat_importances.nlargest(10).plot(kind='barh')

fig.tight_layout()

plt.show()

Support Vector Machine

import numpy as np

import pandas as pd

import matplotlib.pyplot as plt

import seaborn as sns

import matplotlib.pyplot as plt

from sklearn.model_selection import train_test_split

from sklearn.preprocessing import StandardScaler

from sklearn.svm import SVC

from sklearn.metrics import accuracy_score

from sklearn.model_selection import GridSearchCV

from sklearn.metrics import classification_report,confusion_matrix

from sklearn.metrics import roc_curve

from sklearn.metrics import roc_auc_score

from sklearn.model_selection import cross_val_score

from sklearn.model_selection import KFold

df = pd.read_csv("Data_Selected.csv")

df.shape

df.head()

col_names = df.columns

col_names

df['DX'].value_counts()

X = df.drop(['DX'], axis=1)

y = df['DX']

X = StandardScaler().fit_transform(X)

svc=SVC()

X_train, X_test, y_train, y_test = train_test_split(X, y, stratify=y, test_size = 0.3)

parameters = [ {'C':[1, 10, 100, 1000], 'kernel':['linear']}, {'C':[1, 10, 100, 1000], 'kernel':['rbf'], 'gamma':[0.1, 0.2, 0.3, 0.4, 0.5, 0.6, 0.7, 0.8, 0.9]}, {'C':[1, 10, 100, 1000], 'kernel':['poly'], 'degree': [2,3,4] ,'gamma':[0.01,0.02,0.03,0.04,0.05]}]

grid_search = GridSearchCV(estimator = svc, param_grid = parameters, scoring = 'accuracy', cv = 5, verbose=0)

grid_search.fit(X_train, y_train)

print('GridSearch CV best score : {:.4f}\n\n'.format(grid_search.best_score_))

print('GridSearch CV score on test set: {0:0.4f}'.format(grid_search.score(X_test, y_test)))

print('Parameters that give the best results :','\n\n', (grid_search.best_params_))

print('\n\nEstimator that was chosen by the search :','\n\n', (grid_search.best_estimator_))

poly_svc=SVC(C=1000, degree=3, gamma=0.02, kernel='poly') #### output from GridSearch

poly_svc.fit(X_train, y_train)

y_pred=poly_svc.predict(X_test)

print('Model accuracy score with polynomial kernel and C=1.0 : {0:0.4f}'. format(accuracy_score(y_test, y_pred)))

print(confusion_matrix(y_test,y_pred))

print(classification_report(y_test,y_pred))

fpr, tpr, thresholds = roc_curve(y_test, y_pred)

plt.figure(figsize=(6,4))

plt.plot(fpr, tpr, linewidth=2)

plt.plot([0,1], [0,1], 'k--' )

plt.rcParams['font.size'] = 12

plt.title('ROC curve for Predicting a AD conversion classifier')

plt.xlabel('False Positive Rate (1 - Specificity)')

plt.ylabel('True Positive Rate (Sensitivity)')

plt.show()

ROC_AUC = roc_auc_score(y_test, y_pred)

print('ROC AUC : {:.4f}'.format(ROC_AUC))

Cross_validated_ROC_AUC = cross_val_score(poly_svc, X_train, y_train, cv=10, scoring='roc_auc').mean()

print('Cross validated ROC AUC : {:.4f}'.format(Cross_validated_ROC_AUC))

print('Training set score: {:.4f}'.format(poly_svc.score(X_train, y_train)))

print('Test set score: {:.4f}'.format(poly_svc.score(X_test, y_test)))

kfold=KFold(n_splits=10, shuffle=True)

poly_scores = cross_val_score(poly_svc, X, y, cv=kfold)

print('Stratified cross-validation scores with poly kernel:\n\n{}'.format(poly_scores))

print('Average stratified cross-validation score with poly kernel:{:.4f}'.format(poly_scores.mean()))

Decision Tree

import itertools

import pandas as pd

import pylab as pl

import numpy as np

import scipy.optimize as opt

from sklearn import preprocessing

from sklearn.model_selection import train_test_split

import matplotlib.pyplot as plt

from sklearn import svm

from sklearn.metrics import classification_report, confusion_matrix

from sklearn.tree import DecisionTreeClassifier

from sklearn.model_selection import train_test_split

from sklearn import metrics

import matplotlib.pyplot as plt

import pydotplus

import matplotlib.image as mpimg

from sklearn import tree

from six import StringIO

import graphviz

from sklearn.tree import DecisionTreeClassifier, export_graphviz

import matplotlib.pyplot as plt

import pydotplus

df = pd.read_csv("Data_Selected.csv")

df

df.columns

df['DX'].value_counts()

X_Train, X_Test, Y_Train, Y_Test = train_test_split(X, Y, stratify=Y, test_size = 0.20)

DX_Tree= DecisionTreeClassifier(criterion="entropy", max_depth = 3)

DX_TreeFit=DX_Tree.fit(X_trainset,Y_trainset)

FitPredTree= DX_TreeFit.predict(X_testset)

print (FitPredTree [0:5])

print (Y_testset [0:5])

print(confusion_matrix(Y_testset,FitPredTree))

print(classification_report(Y_testset,FitPredTree))

print("DecisionTrees's Accuracy: ", metrics.accuracy_score(Y_testset, FitPredTree))

dot_data = StringIO()

filename = "DXtree_n.png"

featureNames = X.columns

targetNames = df["DX"].unique().tolist()

data = export_graphviz(DX_TreeFit,out_file=None,feature_names= X.columns, class_names=['convert', 'noconvert'], filled=True, rounded=True, special_characters=True)

graph = pydotplus.graph_from_dot_data (data)

graph.write_png(filename)

text_representation = tree.export_text(DX_TreeFit, feature_names=("Left_entorhinal", "Left_lateraloccipital", "Left_middletemporal", "Left_rostralanteriorcingulate", "Left_temporalpole", "Right_entorhinal", "Right_lateraloccipital"))

print(text_representation)
